# Beliefs of People With Patellofemoral Pain About Their Condition and Treatments Before and After Self‐Directed Access to a Web‐Based Education Platform

**DOI:** 10.1002/msc.70165

**Published:** 2025-07-30

**Authors:** Larissa Rodrigues Souto, Danilo De Oliveira Silva, Marcella Ferraz Pazzinatto, Christian John Barton

**Affiliations:** ^1^ La Trobe Sport and Exercise Medicine Research Centre La Trobe University Melbourne Australia; ^2^ Department of Physiotherapy Universidade Federal de São Carlos São Paulo Brazil; ^3^ Discipline of Physiotherapy School of Allied Health Human Services and Sport La Trobe University Bundoora Australia

**Keywords:** anterior knee pain, beliefs, self‐directed learning, web‐based education

## Abstract

**Objectives:**

To evaluate the beliefs of people with patellofemoral pain (PFP) about their condition's development and persistence, and treatment options, before and after self‐directed access to a web‐based education platform.

**Design:**

Pre‐ and post‐intervention trial.

**Methods:**

Fifty‐eight people with PFP completed custom questionnaires (open‐ended and multiple choice) before and 6 weeks after accessing the “My Knee Cap” web‐based education platform. Questions covered beliefs about causes and persistence of pain, effectiveness of treatments, and willingness to undergo surgery. Open‐ended responses were analysed thematically. McNemar's test with Yates correction compared changes between pre‐ and post‐intervention for multiple choice questions.

**Results:**

At baseline and 6 weeks, PFP onset was primarily attributed to loading. At baseline, pain persistence was linked to loading, structure, and muscle impairments, with muscle impairments being replaced by sedentary behaviour at the 6‐week follow‐up. At baseline, most participants (69%–81%) believed that taping, bracing, foot orthoses, and exercises were effective treatment options. Fewer participants (16%–22%) believed that dry needling, injections, ultrasound, and surgery were effective. At 6 weeks, beliefs about the effectiveness of exercise (16% increase, ES = 0.15), injections (13% decrease, ES = 0.44), and ultrasound (15% decrease, ES = 0.50) changed. At baseline and 6 weeks, most participants (93%–95%) believed exercise was more effective than surgery, and 46%–59% were willing to undergo surgery if imaging revealed abnormalities.

**Conclusion:**

Beliefs about the causes and persistence of PFP centred on loading and pathoanatomical factors, remaining largely unchanged after self‐directed web‐based education. Web‐based education may help to promote the benefits of exercise and reduce beliefs that injection and ultrasound are helpful.

## 1 Introduction

Patellofemoral pain (PFP), characterised by pain around or behind the patella during activities that load the patellofemoral joint (Crossley et al. 2016), is one of the most prevalent conditions in general practice (van Middelkoop et al. 2008), as well as in orthopaedic (Bolgla et al. 2008; Feller et al. 2007), and sport settings, with an annual prevalence of approximately 23% of adults in the general population (Smith, Selfe, et al. 2018). PFP is a long‐term persistent condition, with reports that only one‐third of patients are pain‐free one year after diagnosis (Collins et al. 2013), and 57% still have not recovered 8 years post‐treatment with a health professional (Lankhorst et al. 2016).

PFP is a complex multifactorial condition (Barton et al. 2015; Manojlović et al. 2023; Smith, Moffatt, et al. 2018) which makes it difficult for patients to fully understand the many factors that might contribute to the development and persistence of their pain. Qualitative research involving people with PFP indicates that they widely attribute their symptoms onset to inappropriate training or the overuse of the lower extremity (Manojlović et al. 2023). Other qualitative research (Smith, Moffatt, et al. 2018) indicates that people with PFP may also hold strong beliefs about knee structure (e.g., tissue damage) being the key driver of their pain, a belief which may not align with current evidence (Vicenzino et al. 2019).

The complexity of PFP also makes it challenging to identify the most effective treatments. Exercise therapy is widely recognised and used as a key treatment, either as a standalone intervention or in a multimodal approach (Barton et al. 2014, 2015; Collins et al. 2009, 2018; Willy et al. 2019). However, some clinicians still report focussing their treatment on passive interventions, including ultrasound and joint mobilisation/manipulation, with limited evidence of their effectiveness (Barton et al. 2022; Pisani et al. 2022; Willy et al. 2022; Zambarano et al. 2022). Subsequently, patients may struggle to identify the most effective interventions for their condition, prolonging their discomfort and limiting their ability to return to normal activities.

Given potential misconceptions about the development and persistence of PFP symptoms, along with non‐recommended interventions commonly being used, there is a clear need for knowledge translation tools to bridge the gap between current evidence and clinical practice (Barton et al. 2022). The freely available web‐based platform “My Knee Cap” (De Oliveira Silva et al. 2020) was created to support patient education through self‐directed use or in collaboration with a health professional. It was developed by PFP experts, patients and health professionals offering evidence‐based information about PFP and available treatments, including proving guidance for a self‐directed exercise‐therapy programme. Initial evaluation of “My Knee Cap” indicates that one in five participants reported complete recovery after 6 weeks of self‐directed use (De Oliveira Silva et al. 2020). While the platform appears to be associated with positive clinical outcomes, it remains unclear whether it might influence the beliefs about factors associated with pain or effective treatments in those accessing it.

No previous study has evaluated the potential influence of self‐directed access to a web‐based education platform on patients' beliefs about PFP and its treatments. Therefore, we aimed to explore the beliefs of people with PFP about their conditions' development and persistence, and treatment options, before and after 6 weeks of self‐directed access to the web‐based education platform “My Knee Cap”.

## 2 Methods

### 2.1 Study Design

This is a pre‐ post‐intervention trial in which all participants received 6 weeks' access to a self‐directed web‐based education platform, “My Knee Cap” (www.mykneecap.trekeducation.org). The study was reported according to the Transparent Reporting of Evaluations with Nonrandomised Designs (TREND) statement checklist (Des Jarlais et al. 2004).

### 2.2 Participants

Prospective data were derived from two clinical trials (Australian New Zealand Clinical Trials Registry [ANZCTR] 2020; De Oliveira Silva et al. 2020) involving 6‐week self‐directed access to a web‐based education platform for people with PFP. Both trials were registered (ACTRN12618000224224 and ACTRN12620000336987) and approved by the La Trobe University and Ethics Committee (HEC17‐102 and HEC19‐478). The pooled sample comprised 58 participants with PFP, recruited using advertisements at La Trobe University, gyms and on social media (Facebook, blogs, and Twitter). One study (De Oliveira Silva et al. 2020) recruited participants from Melbourne between February 26 and July 1, 2018 while the other (ANZCTR 2020) recruited participants Australia‐wide between March 30, 2020 and August 28, 2023.

Eligibility criteria were based on the consensus statement for clinical examination of people with PFP (Crossley et al. 2016). Participants were required to have anterior or retropatellar pain of at least 3 month duration during at least two or more activities from prolonged sitting, squatting, kneeling, running, ascending and descending stairs, jumping, and landing, and to rate their worst pain severity as at least 30 on a 100‐mm visual analogue scale (VAS) in the previous week. Participants were also required to be able to read and understand English and have internet access. Exclusion criteria included a history of any lower limb surgery, patellar subluxation or dislocation, or ligament or meniscus tears, presence of neurological disease, or having taken oral steroids and/or opiates in the last month (de Oliveira Silva et al. 2018).

### 2.3 Procedures

Eligibility was assessed by a Physiotherapist (> 5 years of clinical experience) at the University's laboratory and/or via an online survey and videoconference (Zoom: Copyright 2012–2023 Zoom Video Communications Inc., version 5.14.11) interview. The online survey and data collection were managed by the Research Electronic Data Capture (REDCap) tool hosted at La Trobe University (Harris et al. 2019, 2009). All participants provided written and verbal consent. Participant baseline characteristics are reported as recommended by the REPORT‐PFP checklist (Barton et al. 2021) and included age (years), body mass (kg), height (m), sex (female, male, intersex, not specified/prefer not to say), symptom duration (months), painful/most painful knee, worst knee pain in the last week collected by using the VAS and self‐reported function and symptoms collected by using the Anterior Knee Pain Scale—AKPS (Kujala et al. 1993).

A novel questionnaire developed by authors D.O.S. and C.J.B. (Perceptions and Experiences Regarding Symptoms, Pain, and Treatment in Patellofemoral Pain—PFP‐PERSPECTIVE questionnaire) was used to evaluate knowledge about PFP and beliefs about its treatments at baseline and at 6‐week follow‐up (Appendix A). The Melbourne cohort (De Oliveira Silva et al. 2020) completed the questionnaire on paper independently at La Trobe Sports and Exercise Medicine Research Centre, La Trobe University, Melbourne, Australia, while the Australia‐wide cohort (ANZCTR 2020) completed it independently through an online survey on REDCap.

After the baseline assessment, all participants received the same intervention, being encouraged to access the web‐based education platform “My Knee Cap” for a period of 6 weeks, with the same outcome measures taken again following access.

### 2.4 Intervention

The web‐based education platform “My Knee Cap” provides education about physical and non‐physical factors associated with the development and persistence of PFP and self‐management of symptoms based on current evidence (Barton et al. 2015; Collins et al. 2018; Lack et al. 2018; Willy et al. 2019) and a self‐directed hip‐ and knee‐targeted exercise‐therapy programme (Barton et al. 2019). All information are provided in plain language, with multimedia resources such as infographics, animated videos, and podcasts provided to optimise engagement and understanding. It was developed with the assistance of a web designer, to be both didactic and engaging. Feedback on content, language, and functionality of the platform was sought during development from two laypersons diagnosed with PFP (i.e., end users), five physiotherapists experienced in managing PFP, and four researchers with a strong track record of publication in PFP. Refinements were made to the website based on their feedback before the commencement of the study.

The version tested in this study contained four main sections:

1. *Understanding your pain.* This section provides information about the diagnosis, prognosis, incidence, and prevalence of PFP, noisy knee, fear of movement, self‐management of exercise load, and pain self‐management.
2. *Treatment options.* This section describes treatment options with established treatment efficacy for pain (taping/bracing, foot orthoses, and exercises), and information about common treatments with inconsistent evidence (knee surgery, ultrasound, and platelet‐rich plasma injections).
3. *Exercise therapy programme.* This section focuses on 4 types of exercises targeting the trunk, hip, and knee muscles, based primarily on a previously published exercise therapy trial (Barton et al. 2019) Videos and images with instructions provide guidance on how to complete and progress each exercise.
4. *Patient stories.* This section presents the stories of 2 patients (1 woman and 1 man), from a private physiotherapy clinic in Melbourne, with PFP who had successful outcomes after engaging in education and exercise therapy.

After the baseline assessment, a Physiotherapist introduced the content of the website during a 30‐min orientation session. In addition, participants were asked to complete one exercise of each type 3 times a week for a period of 6 weeks and access the website as often as needed.

### 2.5 Outcomes

Perceptions and Experiences Regarding Symptoms, Pain, and Treatment in Patellofemoral Pain (PFP‐PERSPECTIVE) Questionnaire.

The initial version of the questionnaire was informed by PFP literature (Barton et al. 2015; Collins et al. 2018; Lack et al. 2018; Willy et al. 2019), and reviewed by two context experts, one clinician and one person with lived experience external to the research team, who had approximately 4 weeks to provide comments on each item of the survey and general comments on potential missing items. Amendments were completed after content revision, and the survey was then piloted with one more person with lived experience of PFP.

All questions were nonrandomised and closed ended (multiple choice), except for the first two questions that included the participants' opinions on why their PFP started and why they still have PFP. The questionnaire consisted of 5 sections with a total of 13 questions, taking approximately 5 min to be completed and covering the following topics:

1. The cause of the onset of PFP (1 question).
2. The reason for the PFP persistence (1 question).
3. Which treatments can help reduce PFP (taping, bracing, knee and hip exercises, foot orthoses, ultrasound, knee surgery, dry needling, and injections) (8 questions). Answers could be ‘yes’, ‘maybe’ or ‘no’.
4. Willingness to undergo surgery based on imaging (magnetic resonance image [MRI] or X‐ray) (2 questions).
5. Which treatment is more effective (surgery or exercise) to treat PFP (1 question).

### 2.6 Data Synthesis and Analysis

All the data were deidentified and exported to Microsoft Excel for synthesis and analysis. Thematic analysis (Braun and Clarke 2023) was applied by L.R.S. to data from open questions 1 and 2, to categorise responses, with checking and refinement completed in collaboration with C.J.B. during three 60‐min meetings. The remaining data were dichotomised for statistical analysis. Regarding question 3, we dichotomised adjunct treatment answers as “yes/maybe” and “no” for taping, bracing and foot orthoses; “yes” and “no/maybe” for dry needling, injections, and ultrasound; for hip and knee exercises, we dichotomised answers as “yes” and “maybe”; and for surgery, we dichotomised answers as “yes” and “no/maybe”.

The data was organised into a 2 × 2 contingency table, and the McNemar test with Yates correction was conducted to determine if there was a statistically significant change in the proportion of responses before and after the intervention. The Yates continuity correction was applied to the chi‐square statistics yielding a more conservative *p*‐value. Effect sizes (ES) were calculated using Phi, and categorised as negligible (< 0.10), small (≥ 0.10), moderate (≥ 0.30) or large (≥ 0.50) (Cohen 1988). The meaningful difference was pre‐determined using a cutoff of 10%, following discussion and consensus among the research team. If a difference equal or greater than 10% was observed, it was considered a clear and meaningful difference. If a difference less than 10% was observed, it was considered uncertain. All analyses were carried out using R software version 4.3.2 and the statistical significance was set at *α* = 0.05.

## 3 Results

**FIGURE 1:**
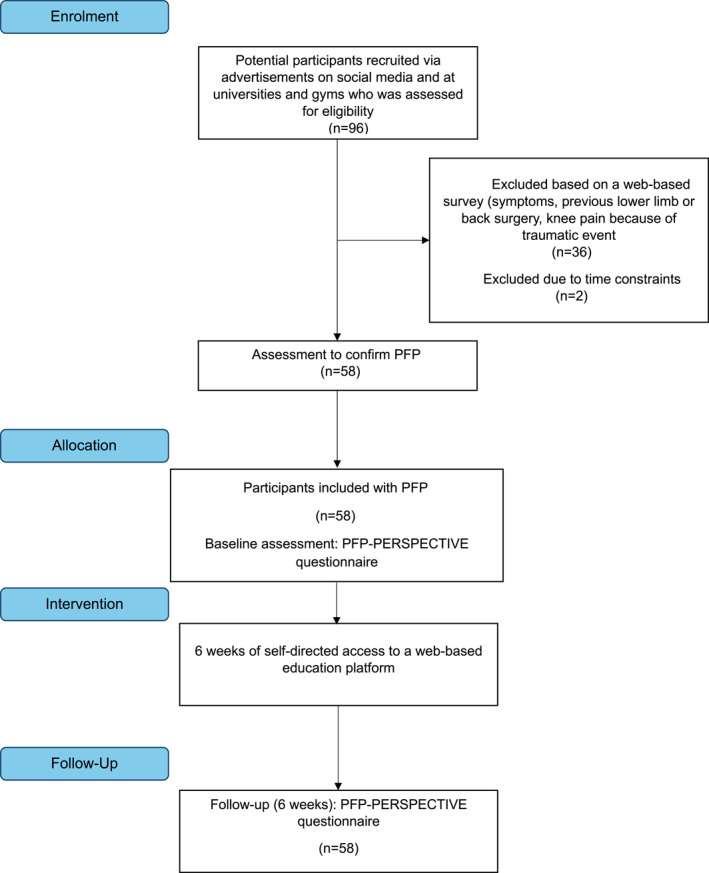
Flow chart of the study.

Ninety‐six potential participants expressed interest in the studies, with 58 meeting eligibility criteria and participating (Figure 1). Baseline demographic characteristics are presented in Table 1.

**TABLE 1:** Demographic characteristics of participants.

	Participants (*n* = 58)
Age (years)	32 ± 5
Sex *n* (%) (female/male)	40 (69%)/18 (31%)
Body mass (kg)	73.0 ± 18.2
Height (m)	1.71 ± 0.10
Body mass index (kg/m^2^)	25.03 ± 5.98
Worst knee pain last week (0–100)	54 ± 20
AKPS (0–100)	75 ± 14
Duration of symptoms (months)	42 ± 71
Painful knee *n* (%)	
Left	11 (19%)
Right	12 (21%)
Both	35 (60%)

*Note:* Data is reported as mean (standard deviation) unless otherwise stated.

Abbreviations: AKPS, anterior knee pain scale; kg, kilogramme; kg/m^2^, kilogramme by square metre; m, metre.

### 3.1 Beliefs About Risk Factors (Question 1)

**FIGURE 2:**
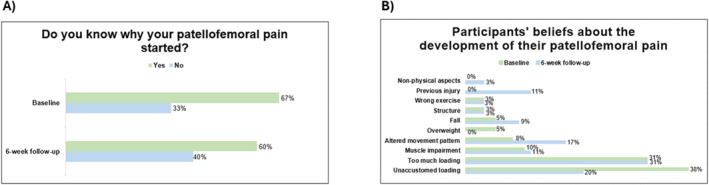
Percentage of participants who believed they knew or did not know why their patellofemoral pain started (A), along with their beliefs about its cause at baseline and at the 6‐week follow‐up (B).

At baseline, 39 participants (67%) believed they knew why their PFP started, reducing negligibly to 35 participants (60%) at 6 weeks (*χ* ^2^ = 0.64; ES: 0.48; *p* = 0.42) (see Figure 2a). A summary of beliefs about the onset of PFP at baseline and 6‐week follow‐up is provided in Figure 2b, with associated quotes provided in Appendix A 1. The most commonly believed reasons for the onset of PFP at baseline and 6‐week follow‐up time points were unaccustomed loading (20%–38%) and too much loading (31%).

### 3.2 Beliefs About Persistence (Question 2)

**FIGURE 3:**
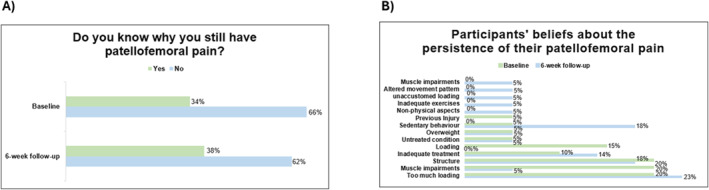
Percentage of participants who believed they knew or did not know why their patellofemoral pain persisted (A), along with their beliefs about its persistence at baseline and at the 6‐week follow‐up (B).

At baseline, 20 participants (34%) believed they knew why they still had PFP, increasing negligibly at the 6 weeks to 22 participants (38%) (*χ* ^2^ = 0.08; ES: 0.55; *p* = 0.77) (see Figure 3a). A summary of beliefs about the persistence of PFP at baseline and 6‐week follow‐up is provided in Figure 3b, with associated quotes provided in Appendix A1. Too much loading (20%–23%) and structure (18%–20%) were the most commonly believed reasons for PFP persistence at both baseline and the 6‐week follow‐up. Muscle impairments (20%) and sedentary behaviour (18%) were also commonly believed reasons at baseline and the 6‐week follow‐up, respectively.

### 3.3 Helpful Treatments (Question 3)

**FIGURE 4:**
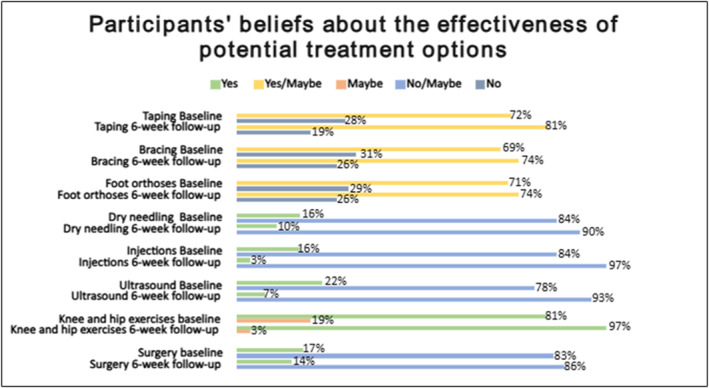
Percentage of participants' beliefs about the helpful treatments at baseline and 6‐week follow‐up.

At baseline, the proportions of participants who believed that taping, bracing, and foot orthoses would or might help (i.e., answered “yes/maybe”) were 72%, 69%, and 71%, respectively. All increased negligibly after 6 weeks compared to baseline (Figure 4). Increases were 9% for taping (*χ* ^2^ = 0.94; ES: 0.19; *p* = 0.33), 5% for bracing (*χ* ^2^ = 0.30; ES: 0.45; *p* = 0.57), and 3% for foot orthoses (*χ* ^2^ = 0.07; ES: 0.39; *p* = 0.78).

At baseline, the proportions of participants who believed dry needling, injections, and ultrasound would or might help (i.e., answered “yes”) were 16%, 16%, and 22%, respectively. All decreased after 6 weeks compared with baseline (Figure 4). Decreases were 6% for dry needling (*χ* ^2^ = 0.30; ES: 0.01; *p* = 0.57), 13% for injections (*χ* ^2^ = 5.14; ES: 0.44; *p* = 0.02), and 15% for ultrasound (*χ* ^2^ = 7.11; ES: 0.50; *p* = 0.007), with only the reductions for injections and ultrasound being statistically significant.

At baseline, 81% of participants believed that knee and hip exercises were effective, and this belief increased significantly by 16% at the 6‐week follow‐up compared to baseline (*χ* ^2^ = 5.81; ES: 0.15; *p* = 0.01) (Figure 4). In contrast, 17% of participants believed that surgery was effective at baseline, and this proportion decreased negligibly by 3% (*χ* ^2^ = 0.08; ES: 0.21; *p* = 0.77) at the 6‐week follow‐up compared to baseline.

### 3.4 Willingness to Undergo Surgery Based on Imaging Alone (Questions 4 and 5)

**FIGURE 5:**
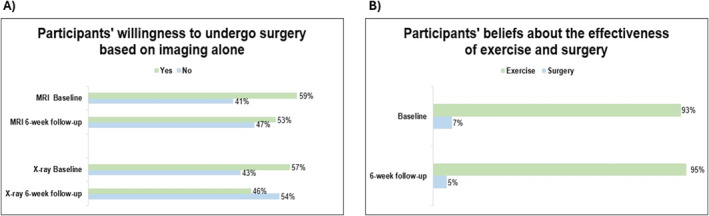
(A) Percentage of participant’ willingness to undergo surgery in case of abnormalities in MRI and X‐ray at baseline and 6‐week follow‐up. (B) Percentage of participant’ beliefs about which treatment is more effective, exercise or surgery, at baseline and 6‐week follow‐up.

At baseline, the proportion of participants willing to undergo surgery if abnormalities were found on MRI and X‐ray were 59% and 57%, respectively. This proportion decreased by 6% (*χ* ^2^ = 0.44; ES: 0.69; *p* = 0.50) for MRI and 11% for X‐ray (*χ* ^2^ = 2.50; ES: 0.67; *p* = 0.11). However, these changes were not statistically significant (Figure 5a).

### 3.5 Surgery Versus Exercise (Question 6)

At baseline, the proportion of participants believing that exercise was more effective than surgery was 93%. This proportion increased negligibly (2%: *χ* ^2^ = 0; ES: 0.55; *p* = 1) at the 6‐week follow‐up compared to baseline (Figure 5b).

## 4 Discussion

Our study provides novel insights into how people with PFP perceive the development and persistence of their pain, alongside their beliefs about potential active, passive and surgical treatment options. Two in three participants believed they knew why their pain started, with a range of different beliefs about factors contributing to the development and persistence of pain shared. The most commonly believed reasons for PFP development at baseline were unaccustomed loading (38%) and too much loading (31%), with participants often describing an abrupt increase in physical activity levels and/or repetitive movements. This finding is consistent with other previous qualitative research involving people with PFP (Manojlović et al. 2023), and clinician beliefs that unplanned or excessive changes in physical activity often trigger PFP symptoms (Curran et al. 2022). While the cause of PFP remains unclear (Lack et al. 2018), these beliefs align with literature proposing that either elevated patellofemoral joint stress (Dye 2005; Powers 2003) or an abrupt increase in physical activity level (Nielsen et al. 2014) can potentially lead to PFP symptoms. At the 6‐week follow‐up, the proportion of participants who claimed knowledge about the cause of their PFP onset decreased negligibly from 67% to 60%. Despite an 18% decrease in the proportion attributing pain development to unaccustomed loading, this factor remained the second most common (20%). Additionally, the proportion believing too much loading was the cause of their pain remained unchanged (31%), indicating that loading factors were considered as the key cause of symptoms by most people with PFP in this study.

At baseline, just one in three participants believed that they knew why their PFP persisted, with the most common reasons related to too much loading (20%), muscle impairments (20%), and structure (20%). These beliefs are variably supported by current evidence (Curran et al. 2022; Dye 2005; Manojlović et al. 2023; Nielsen et al. 2014; Powers 2003). It is possible that high physical activity levels and repetitive movements beyond tissue capacity may generate nociceptive stimuli, and lead to the maintenance of sensitisation (Lack et al. 2018). Evidence supports this assumption, as greater physical activity level has been associated with localised and remote pressure hyperalgesia and poorer self‐reported knee function in female runners with PFP (Pazzinatto et al. 2017). Regarding muscle impairments, people with PFP are reported to have muscle weakness (Lankhorst et al. 2013; Rathleff et al. 2014), reduced rate of force development (Nunes et al. 2018) and altered muscle activity (Barton et al. 2013) of the hip and knee musculature compared to people without knee pain. However, the specific contributions of these impairments to PFP persistence, and the need to address them to alleviate pain and disability remains unclear (Lack et al. 2018).

Participants' beliefs that joint structure (e.g., cartilage damage) contributes to their pain persistence align with previous qualitative findings involving people with PFP who sought secondary care within the UK. (Smith, Moffatt, et al. 2018) This pathoanatomic focus also aligns with most participants in this study expressing a willingness to undergo surgery based on abnormalities identified in their imaging results at baseline (MRI: 59% and X‐ray: 57%). This high proportion willing to undergo surgery based on imaging is consistent with similar research in low back pain(Lemmers et al. 2019), and reflects prevalent societal beliefs where pain is considered to be directly related to tissue damage (Smith, Moffatt, et al. 2018), that only can be fixed by surgery. However, similar to research on back pain (Jensen et al. 1994) and knee osteoarthritis (Culvenor et al. 2019), studies in PFP contradict these notions, with most imaging findings being poorly associated with PFP (van der Heijden, de Kanter, et al. 2016; van der Heijden, Oei, et al. 2016).

At the 6 weeks follow‐up, the proportion of participants believing they knew why their PFP persisted remained similar, including the proportion attributing pain persistence to joint structure. Approximately one in two also continued to express willingness to undergo surgery based on MRI (53%) and X‐ray (46%). This continued high pathoanatomical focus was despite the content of the self‐directed web‐based education platform being dedicated to addressing misinformation about the relationship of structural findings on imaging with pain and disability. This indicates that using self‐directed web‐based resources may not be sufficient to address persistent beliefs focused on the need to fix joint structure. Self‐directed education may also be insufficient to address information about the role of non‐physical factors on PFP persistence, which was covered on the “My Knee Cap” platform. No participants reported any psychological features as being related to their pain at 6 weeks, despite fear of movement, pain catastrophising, anxiety and depression all being reported to be correlated with pain and physical function in people with PFP (Maclachlan et al. 2017). In this case, clinician support may be needed to develop a more holistic understanding of factors that may influence pain and disability in people with PFP.

The proportion of participants attributing their persistent knee pain to muscle impairments decreased from 20% to 5%, while those attributing it to sedentary behaviour increased from 5% to 18%. It is well known that people with PFP exhibit both muscle impairments (Barton et al. 2013; Lankhorst et al. 2013; Nunes et al. 2018; Rathleff et al. 2014) and sedentary behaviour (Glaviano et al. 2017; Piva et al. 2009). The reasons for these observed changes are not clear. The platform emphasises the importance of exercise in managing PFP, particularly the potential benefits of exercise therapy and the need to gradually increase load and participation in physical activity. It is possible that some participants may have felt they had sufficiently resolved muscle impairments through exercises guided by the platform, leading them to believe they were no longer contributing to their persistent PFP. Conversely, participants who felt they had not done enough exercise might have been influenced by this information to perceive sedentary behaviour negatively, contributing to their belief that it could worsen their condition.

Aligning with current evidence (Barton et al. 2015; Collins et al. 2018; Willy et al. 2019), 4 in 5 participants believed exercise was an effective treatment for their PFP at baseline. Additionally, most participants believed that exercise was more effective than surgery at baseline (93%) and 6 weeks follow‐up (95%), despite a high proportion of participants suggesting they would have surgery to address MRI (59%) and X‐ray (57%) findings. Following 6 weeks of self‐directed access to “My Knee Cap, “ the proportion believing exercise was effective increased to 19 in 20 participants, suggesting that self‐directed education including exercise therapy guidance may help to ensure that almost all people with PFP understand the potential value of exercise.

Broadly aligning with evidence and guideline recommendations (Barton et al. 2015; Collins et al. 2018; Willy et al. 2019), most participants believed taping (72%), bracing (69%), and foot orthoses (71%) were effective for treating PFP, while fewer believed dry needling (16%), injections (16%), and ultrasound (22%) were effective. At the 6 week follow‐up, the proportion of participants believing that taping, bracing and foot orthoses were effective negligibly increased to 81%, 74% and 74%, respectively. The proportion of participants believing dry needling, injections, and ultrasound were effective also decreased to 10%, 3% and 7%, respectively. The reductions for injections and ultrasound were both statistically and clinically significant (13% and 15%, respectively). However, the proportion of participants believing in taping, bracing, foot orthoses and dry needling did not appear to change at 6 weeks, indicating that self‐directed education may be insufficient to shift beliefs about these treatments. Therefore, further support with patient education from a clinician may still be needed in relation to adjunct treatments.

### 4.1 Limitations and Directions for Future Research

A key limitation of our study was the pre‐post‐treatment design without a comparator group. This means that it is not possible to determine the effectiveness of self‐directed web‐based education, and whether changes in beliefs identified in our study are the result of accessing the website, or other factors. Additionally, we do not have detailed metrics on the usage and navigation of the website by participants, meaning we cannot determine the influence of these specific factors on outcomes. We also did not evaluate participants' beliefs about other adjunct treatments commonly provided to people with PFP such as electrical stimulation. Future research could evaluate the effectiveness of the “My Knee Cap” platform through randomised controlled trials and analyse the usage and navigation metrics of the site by participants.

### 4.2 Practical Implications

Self‐directed use of the web‐based education resources such as the “My Knee Cap” platform might provide important evidence‐based information about the importance of exercise for people with PFP, alongside communicating the lack of evidence currently supporting injections and ultrasound. However, support from a clinician may be necessary to address strong, persistent beliefs that joint structure is a key factor related to PFP persistence and to educate people with PFP about the potential importance of non‐physical factors. Additionally, clinician collaboration might also be needed to influence beliefs about the effectiveness of other adjunct treatments assessed in our study.

## 5 Conclusion

People with PFP commonly attributed the development of their condition to loading factors, with 6 weeks of self‐directed education having little influence on these beliefs. Persistence of PFP was commonly believed to be associated with too much loading, muscle impairments and structural issues at baseline. Similar beliefs were observed at 6 weeks, with sedentary behaviour also commonly believed to influence persistence following self‐directed web‐based education. Limited changes in beliefs about joint structure influencing pain development and persistence indicate that the self‐directed use of a web‐based platform alone may not be appropriate for people with PFP holding strong pathoanatomical beliefs about the cause and persistence of their pain. However, self‐directed education might help to promote the importance of exercise for PFP management, alongside the lack of current evidence supporting some passive interventions, including injections and ultrasound.

## Author Contributions

**Larissa Rodrigues Souto:** conceptualisation, methodology, data curation, formal analysis, writing – original draft. **Danilo De Oliveira Silva:** conceptualisation, methodology, investigation, writing – review and editing. **Marcella Ferraz Pazzinatto:** conceptualisation, data curation, methodology, investigation, writing – review and editing. **Christian John Barton:** conceptualisation, supervision, methodology, investigation, formal analysis, writing – original draft, review and editing.

## Ethics Statement

Prospective data were derived from two clinical trials (ANZCTR 2020; De Oliveira Silva et al. 2020) involving 6‐week self‐directed access to a web‐based education platform for people with PFP. Both trials were registered (ACTRN12618000224224 and ACTRN12620000336987) and approved by the La Trobe University and Ethics Committee (HEC17‐102 and HEC19‐478). The pooled sample comprised 58 participants with PFP, recruited using advertisements at La Trobe University, gyms and on social media (Facebook, blogs, and Twitter). One study (De Oliveira Silva et al. 2020) recruited participants from Melbourne between February 26 and July 1, 2018 while the other (ANZCTR 2020) recruited participants Australia‐wide between March 30, 2020 and August 28, 2023.

## Conflicts of Interest

The authors declare no conflicts of interest.

## Data Availability

The data that support the findings of this study are available from the corresponding author upon reasonable request.

## Appendix A Perceptions and Experiences Regarding Symptoms, Pain, and Treatment in Patellofemoral Pain—PFP‐PERSPECTIVE Questionnaire.

**Table:**

1) Do you know why your patellofemoral pain STARTED?		( ) Yes	( ) No
Please explain:			
2) Do you know why you STILL HAVE patellofemoral pain?		( ) Yes	( ) No
Please explain:			
3) Which of the following treatments do you think can help your patellofemoral pain?			
Taping your knee	( ) Yes	( ) Maybe	( ) No
Bracing your knee	( ) Yes	( ) Maybe	( ) No
Knee and hip exercises	( ) Yes	( ) Maybe	( ) No
Foot orthoses	( ) Yes	( ) Maybe	( ) No
Ultrasound	( ) Yes	( ) Maybe	( ) No
Knee surgery	( ) Yes	( ) Maybe	( ) No
Dry needling	( ) Yes	( ) Maybe	( ) No
Injection	( ) Yes	( ) Maybe	( ) No
4) Would you be willing to undergo surgery if your MRI report abnormalities?		( ) Yes	( ) No
5) Would you be willing to undergo surgery if your X‐ray report abnormalities?		( ) Yes	( ) No
6) Which is more effective to treat patellofemoral pain?		( ) surgery	( ) exercise

## Appendix B Questions 1 and 2 of the Perceptions and Experiences Regarding Symptoms, Pain, and Treatment in Patellofemoral Pain—PFP‐PERSPECTIVE for the Baseline and 6‐Week Follow‐Up (Table B1, B2).

**TABLE B1:** Themes related to the beliefs about the onset of patellofemoral pain.

Themes	Participant's thoughts
Baseline	
Theme 1: Unaccustomed loading	“Probably after I had kids and started lifting them.” “Because I increased my physical activity level (bike).” “Increased walking activity, and decreased physical activity sometime around 2013–2014.” “Sudden increase in physical activities.” “Since I started doing physical activities daily (going to the gym).” “Increase in physical activities.” “After increase in physical activity (ultimate frisbee + gym) about 2 months ago.” “When I started training regularly in the gym.” “Increased running and plyometric exercises.” “Started after increased kneeling on the ground.” “Hiked 60ks, felt pain 5km into hike, pain seemed to subside but tentative. Pain presented acutely 1 day later.” “It came on after running which was a new activity for me.” “Has been low grade, but became acute suddenly during a run.” “Playing too much football 11 a side without preparation.” “Running.”
Theme 2: Too much loading	“After playing basketball (15 years ago).” “Working in retail where my tasks involved high level of physical activity carrying boxes and kneeling to serve customers.” “Too much squats on gym, many years lifting weight, basically gym.” “Constant squats.” “A prolonged period of heavy squatting.” “Continuous jumping, running and changing of direction.” “Practice sports/use.” “Repetitive weightlifting after coming from endurance sport only.” “Ramping up running speed/mileage too quickly.” “Repeated impact on wrestling mat.” “Working long hours in a row standing up and walking.” “I was playing soccer and jumping in the trampoline.”
Theme 3: Muscle impairments	“I didn't do stretching and because the muscular imbalance.” “9 years old, growth spurt. Muscles developing wierdly.” “Lack of strength & conditioning for work completed.” “Cartilage damage under knee cap, weak quads.”
Theme 4: Altered movement pattern	“While doing leg press machine.” “Running, hyperextended knee whilst running.” “Slightly twisted playing table tennis.”
Theme 5: Overweight	“Because I gained a lot of weight in the last few years.” “Because I am overweight.”
Theme 6: Fall	“Fall during running.” “Fall at work.”
Theme 7: Structure	“Cartilage damage under knee cap, weak quads.”
Theme 8: Wrong exercise	“Because of the wrong exercise.”

6‐week follow‐up	
Theme 1: Too much loading	“Gym.” “Lots of heavy squats.” “Frequent kneeling on the ground.” “Sitting too much at home (cross‐legged) in lockdown, then suddenly taking ballet classes every day.” “Hiking overuse.” “Usage.” “Too frequent squatting and leg press sessions.” “Excessive load playing football and not warming up properly.” “Repeated impact in my teens.” “Running seems to have weakened joint. Exacerbated when twisted playing table tennis.” “Working on my feet for long period of time.”
Theme 2: Unaccustomed loading	“Because I increased my physical activity level.” “After started doing exercises regularly.” “When I started play soccer and jump at gym.” “An increase in physical activities.” “Over‐training, increase in physical activity involving sharp changes of direction.” “Increased running and plyometric exercises at the start of the first lockdown.” “Ramping training too hard.”
Theme 3: Altered movement pattern	“Bad posture and baby lifting.” “Higher hyperextension when running. Treatment increasing quadriceps and hip abduction strength to stabilise knee.” “Lack of stretching and I did exercises incorrectly.” “Running seems to have weakened joint. Exacerbated when twisted playing table tennis.” “Possibly weaknesses in the hips, imbalances in ROM and muscular strength, imperfect technique when performing squats.” “I was doing a knee dominant exercise with weights and let my knee fall inwards and I felt that I had injured it/done it wrong and felt the pain.”
Theme 4: Muscle impairments	“Lack of stretching and I did exercises incorrectly.” “Because I have more muscles outside of my leg than inside.” “Weak glutes/hamstrings.” “Possibly weaknesses in the hips, imbalances in ROM and muscular strength, imperfect technique when performing squats.”
Theme 5: Previous injury	“Because I had an injury and didn't have the right treatment at the time.” “Possible injury sustained 15 years ago?” “I'm told it was patella tendinitis.” “Torn meniscus from gym work.”
Theme 6: Fall	“Falling down the stairs.” “Accidental fall.” “Fall.”
Theme 7: Non‐physical aspects	“Genetic.”
Theme 8: Structure	“Patella mal‐tracking. Cartilage damage. Sore fat pad.”
Theme 9: Wrong exercise	“From the wrong exercise in the gym, and overcoming.”

**TABLE B2:** Themes related to beliefs about the reasons for patellofemoral pain persist.

Themes	Participant's thoughts
Baseline	
Theme 1: Too much loading	“I believe it's because I'm still going to the gym at least 6 days a week.” “Due to the work (cleaner, babysitter).” “Weightlifting form issues and possible inadequate spacing between leg sessions.” “Repeated impact on wrestling mat.”
Theme 2: Muscle impairments	“Probably because the left leg/hip are less developed than right leg/hip.” “Lack of strength & conditioning for work completed.” “Maybe not enough muscle mass and strength.” “Could be overtraining/missing treatment/missing muscle strengthening.”
Theme 3: Structure	“I think I don't have cartilage enough.” “Lax ligaments making my knee unstable.” “Cartledge damage under knee cap.” “I suspect my patellar does not track in its joint correctly and causes inflammation. So, if it were to track better, I would end up with less joint wear.”
Theme 4: Inadequate treatment	“Because I did not treat the right way/time.” “Haven't done anything to fix it! modified my behaviour/walking etc to avoid pain.”
Theme 5: Loading	“Because I still work long hours on my feet.” “Long shifts standing up.” “I'm still loading my knee.”
Theme 6: Untreated condition	“Because I have chondromalacia and it has no treatment.”
Theme 7: Overweight	“Probably due to my weight and because I am not doing exercises due to knee pain.”
Theme 8: Sedentary behaviour	“Probably due to my weight and because I am not doing exercises due to knee pain.”
Theme 9: Previous injury	“As above (I'm told it was patella tendinitis).”

6‐week follow‐up	
Theme 1: Too much loading	“Because I carry a lot of heavy stuff daily and I work all day stand.” “Due to work (with children).” “Still sitting too much during the day (it's better though).” “Running and jumping and other stresses on my knees.” “Still training same activities.”
Theme 2: Sedentary behaviour	“Because I am overweight and don't do physical activities.” “I am not doing exercise and I live a sedentary life.” “Lack of exercise and remedying the area around my knee to better support it.” “Lack of exercise.”
Theme 3: Inadequate treatment	“Haven't done my exercises to fix them.” “I have the knee cap injury, and takes time to heal. I need to have more exercise to have stronger thigh muscle to support my knee.” “Because not enough strengthening.”
Theme 4: Structure	“I think I had cartilage injury.” “Still restrengthening hip muscles to pull it back into alignment.” “As above (patella mal‐tracking. Cartilage damage. Sore fat pad), but pain is less due to stronger VMO and hips.” “Chronic sensitisation of tissues, prone to flare.”
Theme 5: Overweight	“Because I am overweight and don't do physical activities.”
Theme 6: Non‐physical aspects	“Genetic.”
Theme 7: Untreated condition	“Apparently, the injury I have is not one that will heal.”
Theme 8: Inadequate exercises	“Inadequate exercising of hip/glutes.”
Theme 9: Issues not resolved	“Still some issues which haven't been resolved.”
Theme 10: Unaccustomed loading	“Gyms closed and I stepped up my road cycling. I have been doing at‐home exercises but not having weights and equipment has meant that I haven't been keeping up my glute strength as usual.”
Theme 11: Altered movement pattern	“Same reasons as above (possibly weaknesses in the hips, imbalances in ROM and muscular strength, imperfect technique when performing squats), but to a lesser extent.”
Theme 12: Muscle impairments	“Same reasons as above (possibly weaknesses in the hips, imbalances in ROM and muscular strength, imperfect technique when performing squats), but to a lesser extent.”
